# Experiences of Transgender and Gender Nonbinary Medical Students and Physicians

**DOI:** 10.1089/trgh.2019.0021

**Published:** 2019-09-23

**Authors:** Oscar E. Dimant, Tiffany E. Cook, Richard E. Greene, Asa E. Radix

**Affiliations:** ^1^ Department of Medicine, Staten Island University Hospital, Northwell Health, New York, New York.; ^2^ Office of Diversity Affairs, NYU School of Medicine, New York, New York.; ^3^ Department of Medicine, NYU School of Medicine, New York, New York.; ^4^ Department of Medicine, Callen-Lorde Community Health Center, New York, New York.

**Keywords:** transgender, TGNB, LGBTQ, medical students, medical education, physicians

## Abstract

**Purpose:** To explore the experiences of transgender and gender nonbinary (TGNB) medical students and physicians in the United States.

**Methods:** The authors conducted a 79-item online survey using Likert-type and open-ended questions to assess the experiences of TGNB-identified U.S. medical students and physicians. Variables included demographic data, disclosure of TGNB status, exposure to transphobia, and descriptions of educational and professional experiences. Recruitment was conducted using snowball sampling through Lesbian, Gay, Bisexual, Transgender, Queer professional groups, list-servs, and social media. The survey was open from June 2017 through November 2017.

**Results:** Respondents included 21 students and 15 physicians (10 transgender women, 10 transgender men, and 16 nonbinary participants). Half (50%; 18) of the participants and 60% (9) of physicians had not disclosed their TGNB identity to their medical school or residency program, respectively. Respondents faced barriers on the basis of gender identity/expression when applying to medical school (22%; 11) and residency (43%; 6). More than three-quarters (78%; 28) of participants censored speech and/or mannerisms half of the time or more at work/school to avoid unintentional disclosure of their TGNB status. More than two-thirds (69%; 25) heard derogatory comments about TGNB individuals at medical school, in residency, or in practice, while 33% (12) witnessed discriminatory care of a TGNB patient.

**Conclusion:** TGNB medical students and physicians faced significant barriers during medical training, including having to hide their identities and witnessing anti-TGNB stigma and discrimination. This study, the first to exclusively assess experiences of TGNB medical students and physicians, reveals that significant disparities still exist on the basis of gender identity.

## Introduction

Transgender and gender nonbinary (TGNB) people have few protections across the United States, and 30% of respondents in the U.S. Transgender Survey reported being fired or denied a promotion related to being TGNB.^1^ Approximately 1 million U.S. adults (0.4%)^2^ and 1.8% of adolescents^3^ identify as TGNB, and in 2018, 0.7% of matriculating medical students identified as TGNB.^4^ However, we know little about TGNB medical professionals' experiences.

In a 2011 study of 427 LGBTQ (Lesbian, Gay, Bisexual, Transgender, Queer) physicians (1% transgender), physicians reported discrimination, harassment, social rejection, and witnessing discrimination against patients and colleagues, with 65% hearing colleagues disparage LGBTQ patients and 34% witnessing substandard care or denial of care to LGBTQ patients.^5^ In a large 2014 study of medical students, lesbian, gay, and bisexual respondents indicated higher levels of depression, lower levels of perceived social support, and more discomfort with disclosure of sexual orientation than heterosexual students, and a majority rated their campus climates as noninclusive.^6^ A large 2015 study of LGBTQ medical students included 35 self-identified gender minorities, 60% of whom did not disclose their gender at school, mainly due to fear.^7^

Terminology changes but in this article “transgender” describes people whose gender identity does not align with their sex assigned at birth. “Non-binary” includes genderqueer, gender-fluid, agender, and gender nonconforming—that is, those who identify as neither or both male/female or another gender. Table 1 lists additional definitions.

**Table 1.** Terminology and Definitions, United States, 2017

Term	Definition
Transgender woman	A woman who was assigned male at birth
Cisgender woman	A woman who was assigned female at birth
Transgender man	A man who was assigned female at birth
Cisgender man	A man who was assigned male at birth
Gender nonbinary person	A person with a gender identity that is outside of the gender binary of female/male
Transmisogyny	A term coined by Julia Serano to describe the way that transphobia and misogyny interact to create a specific prejudice against transgender women and nonbinary, assigned male at birth people^35^
Cissexism	An ideological system that denies, denigrates, and stigmatizes any form of noncisgender form of behavior, identity, relationship, or behavior and can operate in a similar manner as heterosexism^36^
Intersectionality	A term originally developed by Kimberlé Crenshaw to describe oppression faced by black women; intersectionality, or this interplay of structural oppressions, is also a concept critical to understanding the barriers facing TGNB medical students and physicians^37^

TGNB, transgender and gender nonbinary.

The objective of this study was to investigate the experience of TGNB medical students and physicians. Our hypothesis was that respondents would describe environments in which they encounter discrimination on the basis of their gender identity or expression and do not feel comfortable being out. We expected the results to be worse for respondents who also experience systemic oppression such as racism, ableism, misogyny, and poverty.

## Methods

### Participants and procedures

Participants had to identify as transgender and/or gender nonbinary and also to have been enrolled in a U.S. medical school, be a physician in a U.S. residency, or be licensed to practice in the United States. We conducted a 79-item online survey using Likert-type scales and open-ended questions to assess the experiences of respondents. Demographic variables included gender identity, assigned sex at birth, age, race, and ethnicity. We utilized the Nebraska Outness Scale modified for TGNB individuals, and we asked for descriptions of subjective experiences related to being a TGNB medical student and/or physician and possible incidents of discrimination or harassment.

Recruitment was conducted using snowball sampling through LGBTQ health professional groups and conferences (GLMA: Health Professionals Advancing LGBTQ Equality, Philadelphia Trans Wellness Conference), The Group on Diversity and Inclusion AAMC listserv, and social media (Facebook, Twitter). The survey was in English.

Informed consent was obtained through a page preceding the study, which informed people that their participation was anonymous, voluntary, and they could withdraw at any time and were free to skip any question. It also stated that if they were an NYU student or employee, their academic status, grades, or employment would not be affected by the decision whether or not to participate and record of participation would not be linked to academic nor employment records. The risk of emotional distress was discussed, and the number for the National Suicide Prevention Lifeline was provided. Participants agreed that by proceeding, they were at least 18 years old, had read and understood the consent form, and agreed to participate in this study.

The study was approved by the NYU Langone Health Institutional Review Board as s16-00850.

### Data analysis

Descriptive statistics was analyzed using percentages for categorical variables and mean with standard deviation for continuous variables. For ordinal data on Likert scale, median and interquartile range were used. Data were analyzed using Excel 2013 (Microsoft, Redmond, WA).

## Results

### Demographics

We received 37 eligible responses, of which 1 was excluded to protect their privacy as the sole intersex participant. Characteristics of the population are displayed in Table 2. Over half (21; 58%) were medical students, and 15 (42%) were physicians; of the physicians, 7 (47%) were residents, none were fellows, and 8 (53%) were attendings. Five (63%) attendings had completed a fellowship. Participants were asked if they had left training before completion, which none had.

**Table 2.** Characteristics of Study Population, Transgender and Gender Nonbinary Physicians and Medical Students, United States, 2017

Characteristics of study population (*n*=36)	
	*n* (%)
Professional stage	
Medical student	21 (58.3)
Resident physician	7 (46.7)
Attending physician	8 (53.3)
Gender identity	
Woman or transgender woman, binary	10 (27.8)
Man or transgender man, binary	10 (27.8)
Nonbinary, assigned male at birth	1 (2.8)
Nonbinary, assigned female at birth	15 (41.7)
Sexual identity (multiselect)	
Lesbian	6 (16.7)
Gay	4 (11.1)
Bisexual	5 (13.9)
Straight/Heterosexual	2 (5.6)
Asexual or demisexual	2 (5.6)
Queer	22 (61.1)
Pansexual	3 (8.3)
Race/Ethnicity (multiselect)	
African American	2 (5.6)
Asian or Asian American	2 (5.6)
Hispanic	2 (5.6)
Multiracial	3 (8.3)
White	29 (80.6)
Place of birth	
U.S. born	33 (91.7)
Non-U.S. born	3 (8.3)
Age	
20–24	4 (11.1)
25–29	20 (55.6)
30–34	3 (8.3)
35–39	2 (5.6)
40–44	3 (8.3)
45–49	1 (2.8)
50+	3 (8.3)

### Gender identity

Gender identity was determined using a two-step question^8^ regarding sex assigned at birth (male, female, intersex) and gender (trans male, trans female, male, female, agender, gender-fluid, nonbinary, genderqueer, and/or a term not listed; this was multiselect). Respondents included 10 (28%) binary transgender women and 10 (28%) binary transgender men. There were 15 (42%) nonbinary people assigned female at birth (AFAB) and 1 (3%) nonbinary participant assigned male at birth. If a participant chose only binary terms (female, trans female, male, trans male), they were considered binary. If a participant chose at least one nonbinary term, they were considered nonbinary.

Two (6%) participants identified as heterosexual/straight. Most (22; 61%) identified as queer, followed by lesbian (6; 17%), bisexual (5; 14%), gay (4; 11%), pansexual (3; 8%), and asexual or demisexual (2; 6%). Participants could multiselect sexual orientation labels. The majority were white (29; 81%) with 2 (6%) African American, 3 (8%) Asian American, 2 (6%) Latinx, and 3 (8%) multiracial participants. Most (33; 92%) of the participants were U.S. born, and ages ranged from 23 to 70 with a median of 32.2.

### Education

Data regarding education and specialization are reported in Table 3. Medical schools were distributed throughout the continental United States with roughly one-quarter each in the Northeast, Midwest, South, and West. The year of matriculation ranged from the late 1960s to 2017 with a notable increase beginning in 2010. Of the physicians, nine (60%) were training/trained in primary care (internal medicine, pediatrics, family medicine), six (40%) entered other medical specialties, and two (13%) entered surgical specialties. Specialty choice was free text, and some reported more than one specialty.

**Table 3.** Characteristics of Medical Training Transgender and Gender Nonbinary Physicians and Medical Students, United States, 2017

Medical training	
Geographic distribution of medical schools (*n*=36)	
	*n* (%)
U.S. Northeast Region	8 (22.2)
U.S. Midwest Region	9 (25)
U.S. South Region	10 (27.8)
U.S. West Region	9 (25)
Medical specialization (*n*=15) (free-text response)	
Primary care (internal medicine, pediatrics, and family medicine)	9 (60)
Medical subspecialty	6 (40)
Surgical specialty	2 (13.3)
Outness in training (*n*=36 for whole group, *n*=15 for physicians only)	
Medical school	18 (50)
Residency	6 (40)
Fellowship	0 (0)

### Disclosure

**FIG. 1.**
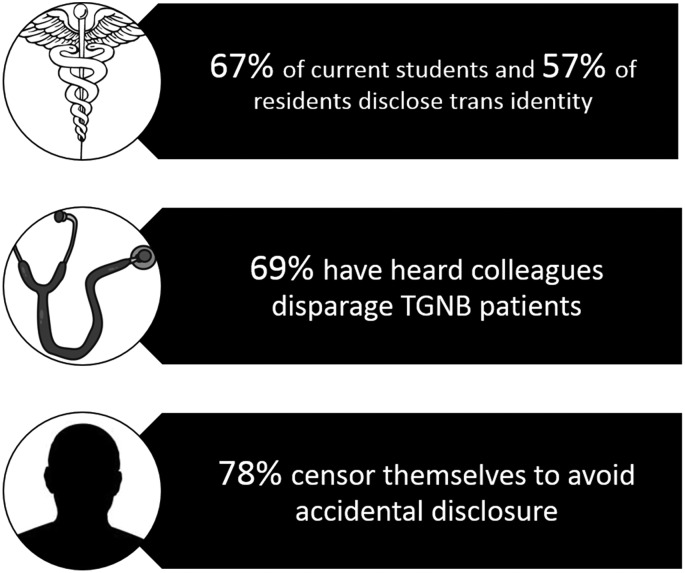
Key results, TGNB physicians and medical students, United States, 2017. TGNB, transgender and gender nonbinary.

Key results concerning disclosure and environment are shown in Figure 1. Eighteen (50%) participants are/were out to their medical schools while six (40%) physicians are/were out to their residency programs and none was out to fellowship programs. Since “outness” is complex, it was framed broadly as being out “to the medical school/residency/fellowship program” at any point, followed by free-text boxes; participants did not differentiate between admission committees, administrators, faculty, and peers, and they spoke of disclosure to these people as a group. When examining current trainees, 14 (67%) students were out to their schools, while 4 (57%, *n*=7) residents were out; none was currently fellows. Three current residents (43%) had been out in medical school. Those who reported nondisclosure cited fears of transphobia, discrimination/harassment, and/or not understanding themselves as TGNB at the time. One participant reported, “I was told by several people that I would not be accepted to medical school if I was out.”

Residents cited similar concerns, and physicians who had completed fellowship spoke of fears of “losing it all” if they came out during fellowship. Participants felt that they had more stress than cisgender counterparts, and one person wrote, “My wellness is quite poor compared to my cisgender co-residents.” Those who trained many years ago mentioned that it was more difficult to be openly TGNB. One physician wrote, “I did not know any other transgender people. There was no one I could talk with until I was over age 40.” Twenty-seven (75%) participants reported that 0–10% of strangers are aware of their TGNB status, which provides context for disclosure choice and vulnerability to discrimination and harassment.

### Transgender health education

Twenty (56%) participants received formal TGNB health education in medical school; data are shown in Table 4. Three participants supplemented their education by creating talks or inviting speakers. Two participants felt positively about their school's education, 12 felt that their education was of fair/good quality but too limited, and 2 felt that their education was of low quality, offensive, and in 1 case “othered and objectified trans people.” Two participants noted that although the TGNB-focused education was of good quality, the remainder of the curriculum undermined it with transphobic language, ideas, and attitudes.

**Table 4.** Characteristics of Transgender and Gender Nonbinary Health Education, Transgender and Gender Nonbinary Physicians and Medical Students, United States, 2017

TGNB health education	
	*n* (%)
How many hours of formal TGNB health education did you receive in medical school? (*n*=34)	
None	16 (47.0)
2 or less	12 (35.3)
3 or more	6 (17.6)
Average (for those who received hours)	2.9 h
How many hours of formal TGNB health education did you receive in residency/fellowship? (*n*=14)	
None	12 (85.7)
2 h or less	0 (0)
3 h or more	2 (14.3)
Average (for those who received hours)	2.75 h

Three (20% of physicians *n*=15; 43% of resident physicians *n*=7) received formal TGNB health education in residency, all of whom were current residents. Two felt that it was of good quality; one was unsure because they were a new resident.

### Environment

For questions concerning discrimination/harassment, participants could answer, “It's complicated.” These data are displayed in Table 5. Seventeen (47%) participants felt that they faced no barriers on the basis of gender identity or expression in medical school applications, 11 (31%) did, and 8 (22%) said “it's complicated.” In residency applications, 7 (50% of 14 who responded) physicians faced no such barriers, 6 (43%) did, and 1 (7%) said “it's complicated.” Two current residents (29% of *n*=7) had faced no such barriers when applying to residency, four (57%) did, and one (14%) said “it's complicated.” Half of those (*n*=8) who had applied to physician jobs felt that they faced no such barriers, while half did. Barriers included inappropriate interview questions, outright hostility, lacking mentorship, and not being able to use a preferred/lived name.

**Table 5.** Discrimination and Harassment, Transgender and Gender Nonbinary Physicians and Medical Students, United States, 2017

Experiences of discrimination or harassment (*n*=36 if medical students included, *n*=15 for physicians-only groups)			
	Yes, *n* (%)	Unsure, *n* (%)	No, *n* (%)
Did you face barriers on the basis of TGNB status when applying to			
Medical school	11 (22.2)	8 (30.6)	17 (47.2)
Residency/fellowship programs	6 (42.9)	1 (7.1)	7 (50)
Jobs as a physician	4 (50)	0	4 (50)
While at your training program or in practice as a physician, have you			
Heard derogatory comments about TGNB individuals	25 (69.4)	3 (8.3)	8 (22.2)
Witnessed discriminatory care or a refusal to provide care to a TGNB patient	12 (33.3)	3 (8.3)	21 (58.3)
Witnessed discriminatory treatment of a TGNB peer, colleague, or superior	8 (22.2)	1 (2.8)	27 (75)

Only eight (22%) participants were able to say that they have not heard derogatory comments about TGNB individuals. Comments were about specific patients, sometimes with colleagues and superiors mocking the TGNB patient during sign-out, intentional misgendering, or using dehumanizing language. One person reported that transphobic comments were about the participant themself. One student reported, “I faced overt verbal abuse and discrimination by nurses, med techs, residents, and attendings with no recourse, no protections or support by the med school, and no mentors or advocates.” Another said, “On rotations I have experienced everything from listening to other staff/residents joking about how ‘freakish’ a trans patient is to experiencing a transgender patient being assaulted by a resident. I do not feel safe being out in my 3rd/4th year rotations at all.” Twelve (33%) participants witnessed discriminatory care or refusal to provide care to a TGNB patient, 3 (8%) were unsure, and 21 (58%) had not witnessed this. One student witnessed a resident perform a nonindicated genital examination on a transgender female patient against her will and specifically because the patient was transgender, and the student described what it was like to witness this as a TGNB person who personally feared sexual assault fueled by transphobia/cissexism. One physician reported, “[I witnessed] derogatory jokes about trans patients, misgendering, misnaming, speculating about their sex/gender, [and] judging patients' gender identity and ‘successfulness' of their transition based on how they visually looked.”

Eight (22%) participants witnessed discriminatory treatment of a TGNB peer, colleague, or superior, and 28 (78%) participants censored speech and/or mannerisms at least half of the time while at work/school to avoid unintentional disclosure of their TGNB status. Participants reported a lack of mentors, although one reported, “I work with a trans doctor weekly and this experience has been amazingly formative. […] Before this last year, I had never met any trans physicians and so was having a hard time imagining what my life might look like and if it was feasible to be trans and a provider.”

Five participants commented that their socioeconomic backgrounds were lower than those of colleagues, which put them at risk for classism. Six participants had physical (five), learning (two), and/or psychiatric (one) disabilities, which put them at risk for ableism. Three participants faced racial microaggressions; one student of color felt that since they were perceived as white, faculty gave them “the time of day” more so than for students perceived as people of color.

## Discussion

### Demographics

Study participants were predominantly white transgender men or nonbinary AFAB people, consistent with national data revealing educational access inequities disproportionately affecting transgender women and people of color.^1^ The results also demonstrated that participants have diverse sexual orientations with only 6% heterosexual, consistent with prior research.^1^ The disproportionate whiteness of the sample is consistent with national data; in 2018, 61.7% of matriculating medical students were white,^3^ which likely reflects wider social disparities.

### Education

The physician participants predominantly chose primary care (nine; 60%). Although this study did not investigate motivations, LGBTQ people may perceive primary care as more inclusive and inclusivity as important.^9^

In 2011, a survey revealed that an average of 5 h in medical school was devoted to LGBTQ health, and transgender topics were least likely to be covered.^10^ Although the majority of participants did receive formal TGNB health education in school, they usually regarded it as insufficient in quality or quantity. In residency, almost no physicians in this sample received formal TGNB health education. This is consistent with documented inadequacies in primary care, emergency medicine, and psychiatry residencies, including a 2019 study of over 1000 internal medicine residents that found that trainees at all levels had similarly low levels of knowledge of sexual and gender minority health.^11–17^

### Environment

It was difficult for respondents to assess application processes, and a minority was able to say that they did not face barriers on the basis of their TGNB identity. Participants lacked mentorship, application forms did not reflect their identities, they fielded offensive interview questions, and they encountered ignorance throughout interview processes. Even after acceptance, participants entered environments with high levels of anti-TGNB bias, as described above.

Perhaps most concerning is the lack of improvement since 2011, when 65% of LGBTQ physicians had heard colleagues disparage LGBTQ patients and 34% had witnessed substandard care or denial of care to LGBTQ patients.^5^ In this sample 69% of participants heard colleagues disparaging TGNB patients, and 33% witnessed discriminatory care or refusal to care for a TGNB patient. The AAMC has published core competencies and a guide for improving climate and curriculum for people who are LGBTQ, gender nonconforming, or have differences in sex development.^18^ However, the data discovered here show no trend toward improvement. The data of 2018 show that LGBTQ health care professionals may face LGBTQ-related stress and being out may result in lack of promotions or tenure and social ostracization.^19^ One physician in this study was asked to leave their practice, and multiple participants reported added social stress upon coming out.

Transmisogyny may result in transfeminine people becoming more frequent targets of violence and suffering more severe violence than transmasculine people,^20,21^ and anecdotally, transfeminine people may be more easily identified as TGNB, although medical support of TGNB children is changing this. Three-quarters of this sample were not easily identifiable as TGNB by strangers, but while “invisibility” decreases the risk of external minority stress, it increases the risk of internal minority stress by requiring that people conceal their identities and isolate themselves. The large proportion of participants who were afraid to be “visible” is concerning; trainees work in close quarters for long hours, and significant bonding and support networks form.^22^ Students are particularly vulnerable as team members with the least power, in addition to being dependent on feedback and scores to move forward. Residents are more established but also continue to be vulnerable as trainees. Gender identity and expression is a core part of self and feeling free to speak about oneself is part of wellness; coming out in a supportive environment may even protect against psychopathologies and chronic physiologic stress.^23^ There is substantial evidence that sexual and gender minority exposure to prejudice, stigma, and minority stress is associated with increased risk of chronic disease such as cardiovascular disease, mental illness, and adverse health outcomes up to and including increased mortality.^24–30^ In addition, two-thirds of physicians in Eliason's group thought that coming out to patients enhanced trust and the relationship.^5^

Feeling pressure to remain closeted may harm TGNB physicians' careers in material ways. Out physicians receive more referrals of LGBTQ patients, are seen as experts on LGBTQ health, and are asked to speak about LGBTQ topics.^5^ In a group of 250 LGBTQ (4.5% TGNB) health care professionals and trainees, 79.4% were involved in LGBTQ-related educational, research, service, or clinical activities and 81% of trainees were interested in academia, although only 47% of professionals held faculty appointments; lack of mentorship/networking and noninclusive environments were barriers, which this study reinforces.^31^

Assessing medical education and climates through the lens of TGNB students and physicians may also benefit patients. Providing greater access to TGNB providers is one approach to eliminating TGNB health inequities, since access to physicians who share salient identities with their patients increases patient trust, satisfaction, and intention to adhere to treatment.^32–34^

Some participants (*n*=13) reported multiple intersecting identities that have historically and continue to be oppressed. This intersectionality likely produces compounding negative effects due to systemic oppression. In free-text responses participants who are people of color, have disabilities, and/or come from low socioeconomic statuses spoke about racism, ableism, and classism that they experienced; this information suggests that improvement efforts must include a holistic view of societal injustice to achieve substantive change.

Strengths of this study include the research team's personal connections with the TGNB community that informed the survey questions and facilitated recruitment.

Limitations include that the investigators were unable to enroll respondents who left medical school or residency. The recruitment method (through social sites and LGBTQ organizations) may also have selected those who are more engaged in online community. A final limitation is the small sample size (36); however, this is still the only study to specifically investigate the experiences of TGNB medical students and physicians, and further research is warranted and needed.

## Conclusion

This study underscores the barriers that TGNB medical students and physicians face throughout the continuum of their careers, from medical school admissions through undergraduate and postgraduate medical training and subsequent employment. In many cases individuals hid their identity due to fear of discrimination, substantiated by witnessing high levels of anti-TGNB stigma and discrimination. Both medical school and residency curricula have not sufficiently included TGNB health topics nor changed transphobic attitudes. Even attending physicians reported environments that continued to perpetuate stigma and discrimination.

TGNB medical students and physicians in this study report adverse experiences but continue to persevere and pursue their goals of becoming physicians and caring for those in need; it is time for medical schools and hospitals to undertake the work of ensuring a safe learning and working environment for all, including those who are TGNB.

## Presentations of This Abstract

AAMC Annual Meeting, Texas, November 2018; GLMA Annual Conference on LGBTQ Health, Nevada, September 2018; NYACP Chapter Annual Scientific Meeting, New York, June 2018; New York University Langone Health LGBTQ Health Awareness Week, New York, March 2018; GLMA Annual Conference on LGBTQ Health, Pennsylvania, September 2017; GLMA Annual Conference on LGBTQ Health, Missouri, September 2016.

## Abbreviations Used

AFAB: assigned female at birth
LGBTQ: Lesbian, Gay, Bisexual, Transgender, Queer
TGNB: transgender and gender nonbinary
